# The effects of warming and nitrogen addition on ecosystem respiration in a Tibetan alpine meadow: The significance of winter warming

**DOI:** 10.1002/ece3.4484

**Published:** 2018-09-05

**Authors:** Ning Zong, Shoubao Geng, Cheng Duan, Peili Shi, Xi Chai, Xianzhou Zhang

**Affiliations:** ^1^ Lhasa National Ecological Research Station Key Laboratory of Ecosystem Network Observation and Modelling Institute of Geographic Sciences and Natural Resources Research Chinese Academy of Sciences Beijing China; ^2^ University of Chinese Academy of Sciences Beijing China

**Keywords:** alpine meadow, ecosystem respiration, N addition, simulated warming, Tibetan Plateau

## Abstract

In recent decades, global warming has become an indisputable fact on the Tibetan Plateau. Alpine ecosystems are very sensitive to global warming, and the impact may depend on the degree of atmospheric nitrogen (N) deposition. The previous studies have paid more attention to year‐round warming, but the effect of winter warming has been unstudied. In this study, a manipulative experiment was conducted, consisting of warming and N addition. It was carried out since 2010 in an alpine meadow, and three types of warming treatments were set up: no warming (NW), year‐round (YW), and winter warming (WW). Warming significantly increased air and soil temperature, but decreased soil moisture. Under no N addition, YW showed significantly decreased ecosystem respiration (Reco) in 2012, and WW decreased Reco in 2014. Under N addition, neither YW nor WW had significant effects on Reco, indicating that N addition compensated the negative effect of warming on Reco. Annually, YW and WW decreased ecosystem carbon (C) emissions, and the extent of the reduction was even larger under WW. Under no N addition, both YW and WW significantly decreased aboveground biomass. Moreover, especially under no N, YW and WW significantly decreased soil inorganic N. WW also had negative effects on soil microbial biomass C. Structure equation modeling showed that soil moisture was the most important factors controlling Reco, and soil inorganic N content and microbial biomass C could explain 46.6% and 16.8% of the variation of Reco. The findings indicate that soil property changes under warming had substantial effects on ecosystem C efflux. The inhibitory effects of winter warming on ecosystem C efflux were mainly attributed to the decline of soil N and microbial biomass. Thus, the effects of winter warming on ecosystem C emissions in this semiarid alpine meadow are not as serious as expected and largely depend on N deposition.

## 1 INTRODUCTION

Global warming and atmospheric nitrogen (N) deposition are important aspects of global change. The IPCC (2007) reported that global temperatures displayed significant seasonal differences, especially in the winter for high‐latitude and high‐altitude areas. Under future climate change scenarios, this asymmetric warming trend will be even more pronounced (Kreyling, 2010). N is an important element limiting the productivity of terrestrial ecosystems (Elser et al., 2007; LeBauer & Treseder, 2008; Wedin & Tilman, 1996). The amount of global N deposition increased more than three times in the last century (Gruber & Galloway, 2008; IPCC, 2007) and is projected to increase by two to three times by the end of this century (Lamarque et al., 2005). N deposition increases have seriously affected the structure and function of terrestrial ecosystems (Galloway et al., 2004), but the extent to which the effects on terrestrial ecosystems interact with warming is unclear (Dormann & Woodin, 2002). Although many studies have been conducted on warming and N deposition in terrestrial ecosystems, these studies have mainly been single factor experiments over a short time period. Furthermore, studies on the effects of asymmetric seasonal warming on ecosystems are still lacking (Hutchison & Henry, 2010; Turner & Henry, 2009). Therefore, in order to obtain a deeper understanding of the impacts of global change on terrestrial ecosystems, a comprehensive study on ecosystem carbon (C) emissions, in response to asymmetric seasonal warming and increased N deposition, is urgently needed.

Elevated temperature can affect the ecosystem N cycle. With sufficient soil water content (Sw), warming can stimulate the N mineralization rate (Rustad et al., 2001), and plant productivity increases resulting from warming may increase N demand for plant growth (An et al., 2005). In addition, warming can potentially increase ecosystem N losses during the winter period, particularly in ecosystems that frequently experience soil freezing and thawing events (Fitzhugh et al., 2001). In these ecosystems, increased N mineralization rates during winter time, when plants are largely in their dormant period, coupled with soil freezing and thawing changes caused by snowpack decline (Groffman et al., 2001), can lead to N loss increase from leaching (Joseph & Henry, 2008; Yanai, Toyota, & Okazaki, 2004). In addition, in alpine meadows, winter warming also affected the seasonal partitioning of soil N by plants and soil microorganisms, which can decrease soil nutrient release for plant growth in the early growing season (Edwards & Jefferies, 2013; Jaeger, Monson, Fisk, & Schmidt, 1999). These increased N losses over the winter and the decrease in nutrient release in the early growing season may limit primary productivity increase in response to experimental warming. The impacts of winter warming on ecological processes may be largely different from annual warming, as winter climate may play a critical role in N retention and other important nutrients (Kielland, Olson, Ruess, & Boone, 2006; Schimel, Bilbrough, & Welker, 2004). Thus, studies on the specific effects of winter warming on ecosystems are very important.

Recognition of the controlling factors is critical for accurately estimating C emissions. Illustrating the controlling factors for ecosystem respiration (Reco) is vital for estimating C balance and understanding the mechanisms of ecosystem CO_2_ emissions under future global change scenarios. Generally, temperature is one of the important factors which affect Reco. However, in arid or semiarid areas, the relationship between Reco and temperature may be confounded by other environmental factors, such as soil water availability (Jiang et al., 2013; Tang, Baldocchi, & Xu, 2005; Xu & Qi, 2001). The direct impact on Reco is that soil moisture affects the physiological activities of plant roots and soil microorganisms, and the indirect impact is that soil moisture affects the transfer process of the substrates and O_2_ for respiration (Luo & Zhou, 2006). Warming and N addition also affects plant production and soil properties, which inevitably causes ecosystem C efflux to change, as plant production and soil microorganisms are important sources of ecosystem C efflux. However, whether or not the controlling factors change under different warming treatments and N addition is still unclear.

Accounting for more than 60% of the area of the Qinghai‐Tibet Plateau, alpine meadows are the basis for maintaining forage production and the development of livestock husbandry and are very sensitive to global climate change (Chen et al., 2013). In recent decades, global change has already imposed pronounced effects on ecosystem C and N cycles in alpine grasslands (Chen et al., 2013). Meteorological observation showed that, over the last several decades, asymmetric seasonal warming (with the most significant warming in winter) was very notable on the Tibetan Plateau (Li, Yang, Wang, Zhu, & Tang, 2010; Liu & Chen, 2000). Relative to other regions, this area is projected to experience a large degree of climate warming in the next several decades (IPCC, 2007). However, studies of soil N dynamics in the winter for the Tibetan Plateau have nevertheless received little attention. Therefore, the recognition of controlling factors on the C cycle under winter warming and increased N deposition can help predict the response as well as the feedback to global change.

In this study, we investigated how warming and N addition regulating ecosystem C efflux in an alpine meadow ecosystem and isolated the specific effect of winter warming from year‐round warming. We arranged the experiment in a factorial design with N addition, and we used open‐top chamber devices (OTCs) to generate warming effects either for year‐round or only winter treatment. We hypothesized that warming and N addition would have interactive effects on Reco. Based on the results that winter warming could increase soil N losses, and that the alpine ecosystem is N‐limited, we predicted that warming would increase ecosystem C efflux under N addition treatment. We also predicted that it would restrict ecosystem C efflux under the no N addition treatment. In addition, winter warming may decrease plant production and ecosystem C efflux, as winter warming can increase soil N loss but is not affected by the warmer temperatures over the summer.

## 2 MATERIALS AND METHODS

### 2.1 Study area

This study was conducted in an alpine meadow in the Damxung grassland station, approximately 3 km north of Damxung County, Tibet Autonomous Region, China. Damxung County is in the central part of the southern region of the Tibetan Plateau (91°05′E, 30°29′N). The altitude is 4,333 m above sea level, and the climate is a semiarid continental type. The long‐term mean annual temperature is 1.3°C, and the precipitation is 477 mm, with 85% of precipitation occurring from June to August (Shi et al., 2006; Zong et al., 2014). The soil is classified as a meadow soil with sandy loam; the depth is approximately 0.3–0.5 m (Shi et al., 2006), and it is composed by 67.02% sand, 18.24% silt, and 14.74% clay (Zong et al., 2014). The surface soil bulk density is 1.29 g/cm^3^. Detailed soil properties can be found in Zong et al. (2014). The plant community cover is approximately 30%–50%, with *Kobresia pygmaea* C.B. Clarke var. *pygmaea*,* Carex montis‐everestii*, and *Stipa capillacea* Keng as dominant species. In addition, the meadow has also been invaded by *Anaphalis xylorhiza* due to overgrazing degradation. The total atmospheric N deposition at this site is approximately 10 kg N ha^−1^ year^−1^ (Zong et al., 2016).

### 2.2 Experimental design and microclimate monitoring

Field manipulations consisted of three warming treatments, year‐round warming (YW), winter warming (WW), and no warming (NW), were crossed with N addition treatment. The N addition rate was 40 kg N ha^−1^ year^−1^, roughly equaling four times greater than the background N deposition rate. The warming and N addition treatments were organized in a randomized block design with five replicates for each treatment. Following the methods of the International Tundra Experiment, passive warming was used with open‐top chambers (OTCs). The OTCs, with a 100 cm diameter in the top opening, 140 cm diameter in the bottom, 40 cm in height, and a bottom area of 1.54 m^2^, were made of 3‐mm‐thick polycarbonate plastic. This material has high solar transmittance in visible and ultraviolet wavelengths (approximately 90%) (De Frenne et al., 2010). We conducted winter warming treatments from 28 September 2012 to 15 May 2013 and again from 30 September 2013 to 17 May 2014. In the N‐added plots, N fertilizer (ammonium nitrate, NH_4_NO_3_) was sprayed as an aqueous solution, twice during the growing season. The first half was added during the early growing season in early June and the remaining half split in early August.

We set up the experiment in July 2010 and synchronously monitored air temperature, soil moisture, and temperature at 5 cm depth by a HOBO weather station (Onset Inc., Bourne, MA, USA) at half‐hour frequency. Rainfall data were obtained from the national Damxung weather station (4,288 m a.s.l., 3 km away from study site) and downloaded from the China Meteorological Data Sharing Service System (http://cdc.cma.gov.cn).

### 2.3 Measurement of ecosystem respiration

Ecosystem respiration (Reco) was measured from June to September in 2012, 2013, and 2014, using a measuring system LI‐8100 (LI‐COR Biosciences, Lincoln, NE, USA). The LI‐8100 system was attached to a chamber, 20 cm in diameter and 4.07 L in volume, and linked to a gas analyzer. At least 1 month before each measurement, one PVC collar (20 cm in diameter and 5 cm in height) was randomly inserted into soil to a depth of approximately 3 cm in each plot for Reco measurement. Plants in the collar were left intact, so that the measured respiration could represent Reco (composed by above and belowground components) (Jiang et al., 2013; Lin et al., 2011). In each PVC collar, Reco was measured from the linear rate of CO_2_ accumulation within the sealed cylindrical headspaces. During the Reco measurement process, PVC collars were covered by a removable lid that contained an opening with a CO_2_ sensor. After closing the lid, CO_2_ monitoring within the cylindrical headspace lasted for 1.5 min. Ecosystem CO_2_ flux rates were calculated as a linear CO_2_ increase using the 1‐s readings during the 1.5‐min closure time, with the initial 15‐s mixing time after lid closure discarded in a LI‐8100 file viewer application software, (Heinemeyer et al., 2011; Zong et al., 2017). Reco measurement was conducted approximately three times in each month, at an approximately 10‐day interval from June to September during every growing season.

### 2.4 Measurement of plant production and soil properties

Plant aboveground biomass was estimated using a nondestructive method (Wang et al., 2012; Zong, Chai, Shi, & Yang, 2018). Briefly, for each plot in mid‐August of 2012, 2013, and 2014, plant community height and cover were measured using a 50 × 50 cm quadrat divided into twenty‐five 5 × 5 cm subquadrates. In 2012, we carried out this process in a nearby alpine meadow by measuring the community height and cover, harvesting, oven‐drying, and weighing. The following equation was used to simulate the relationship between aboveground biomass (AGB) and vegetation height (H) and cover (C): AGB = 0.269 + 3.466C + 0.752H (*R* ^2^ = 0.658, *p *<* *0.001, *N* = 80). Details of this estimation method can be found in Zong et al. (2018). After plant material collection in mid‐August, a soil drill sampler (5 cm in diameter) was used to take 0‐ to 20‐cm soil samples, which were immediately passed through a 2‐mm sieve to pick out plant roots. These root samples were washed, separated, oven‐dried at 65°C for 48 hr and weighed. The sieved soil was then mixed as a composite sample and refrigerated in the laboratory. ${\text{NO}}_{3}^{-}$–N and ${\text{NH}}_{4}^{+}$–N in the composite soil sample were extracted using 2.0 mol/L KCl, filtered, and analyzed using a continuous flow analyzer (AA3, SEAL Analytical, Norderstedt, Germany). The sum of ${\text{NO}}_{3}^{-}$–N and ${\text{NH}}_{4}^{+}$–N represented soil inorganic N content (SIN).

Soil microbial biomass carbon (SMC) was measured by the chloroform fumigation‐extraction method (Vance, Brookes, & Jenkinson, 1987). Briefly, fumigated and unfumigated soil samples were extracted with 0.5 mol/L potassium sulfate (K_2_SO_4_) and filtered through a 0.45‐μm membrane. The extractable organic C was determined by a liquiTOC II analyzer (Elementar Co., Hanau, Germany) and converted to SMC using conversion coefficients of 0.45 (Xu et al., 2010).

### 2.5 Statistical analysis

A repeated‐measure ANOVA was applied to assess the effects of warming and N addition on ecosystem CO_2_ flux. For monthly average ecosystem CO_2_ flux, plant biomass, SIN, and SMC, a two‐way ANOVA was used to test the differences between different treatments and followed by Duncan's test for multiple comparisons. Regression analyses were also used to test the relationships between ecosystem CO_2_ flux and Sw, soil temperature, plant aboveground and belowground biomass, SIN, and SMC in different years. The average growing season Reco was averaged by daily respiration data measured during each growing season. Total C emissions from the entire growing season were the sum of the monthly C emissions. A previous study in the same ecosystem found that the proportion of C released during the growing season was 97.4% of the total annual amount (Zhang, 2005). All the analyses were performed in SPSS 16.0 (SPSS for Windows, Version 16.0, Chicago, IL, USA), and all the figures were produced using Origin Pro 8.0 (OriginLab Corporation, Northampton, MA, USA).

Structure equation modeling (SEM) was also used to evaluate the direct and indirect effects of different environmental variables on Reco. Based on the theoretical knowledge of major environmental factors regulating the variations of ecosystem CO_2_ efflux, a path model was developed to evaluate the interactive relationships between Reco, Sw, SIN, SMC, and AGB. The adequacy of this model was evaluated by the chi‐square test and Akaike information criterion (*AIC*). Nonsignificant chi‐square tests (*p* > 0.05) and a low *AIC* value suggested that the model could be accepted as a potential explanation of the observed covariance structure (Grace, 2006). Based on the *AIC* values, nonsignificant pathways were removed to improve the model adequacy. Eventually, the final model was relatively strong: *χ* ^2^ = 1.044, probability level = 0.307, RMSEA = 0.019, and CFI = 1.00. Furthermore, in this path model, *R*‐squares for Reco were relatively high. The SEM was performed using Amos 17.0 (SPSS Inc.).

## 3 RESULTS

### 3.1 Variations of meteorological factors

**Figure 1:**
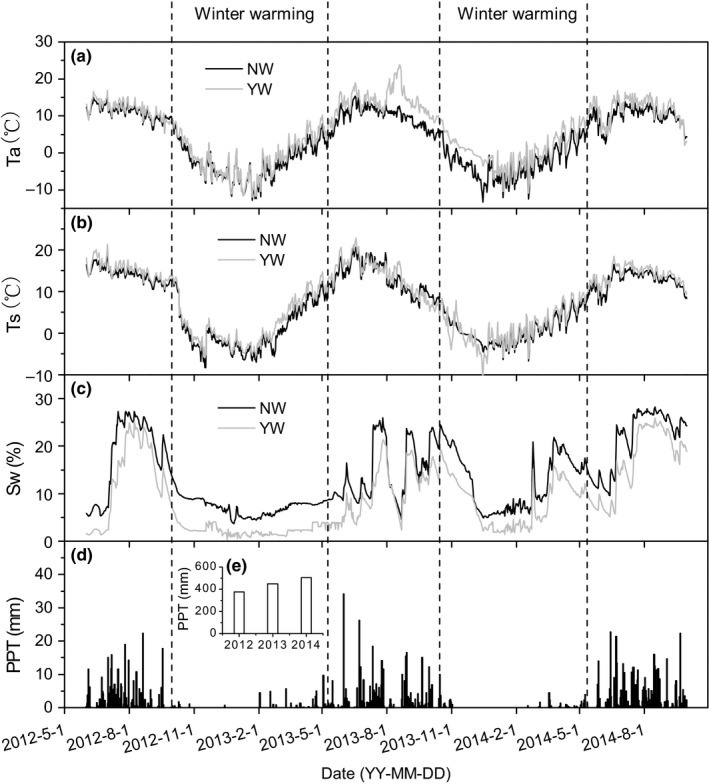
Seasonal dynamic of air temperature (*T*_a_, a), soil temperature (*T*_a_, b), and soil water content (Sw, c) at 5 cm depth in warming (YW, gray line) and ambient (NW, black line) conditions, along with rainfall distribution from June 2012 to September 2014 (d). The insert panel (e) showed the total rainfall from 2012 to 2014

Simulated warming significantly increased air temperature and soil surface (0–5 cm) temperature, but decreased soil moisture (0–5 cm) (Figure 1a–c). From June 2012 to September 2014, the OTC warming devices increased air and soil temperature by 1.6 and 1.4°C, respectively, while reducing soil moisture by 4.7% (v/v). Winter warming can lead to an increase approximately 1.6°C above ambient condition. Compared to the ambient conditions, the warming devices created a warmer but drier conditions.

Annual precipitation was 376.8, 447.3, and 504.1 mm in 2012, 2013, and 2014, with 312.8, 332.5, and 431.9 mm during each growing season (from June to September), respectively. The precipitation was 66.4 and 105.8 mm during the two winter warming periods (Figure 1d).

### 3.2 Seasonal variations of ecosystem respiration and annual ecosystem CO_2_ efflux

**Figure 2:**
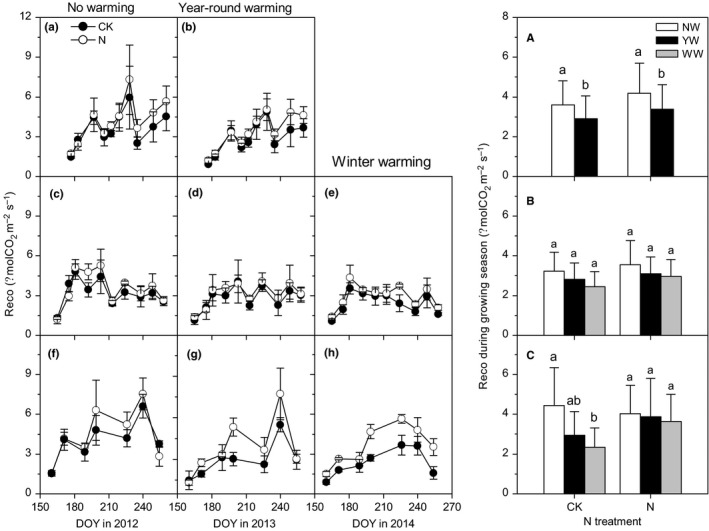
Effects of warming and N addition on seasonal variations and average ecosystem respiration during growing seasons in 2012 (A, B, a), 2013 (C–E, b), and 2014 (F–H, c). Different low case letters under the same N level represented significant differences among warming treatments

Statistical analysis showed that Reco presented significant seasonal variations (Table 1, *p* < 0.001). However, the timing of peak values varied between years, occurring at mid‐August (DOY 228), late‐July (DOY 203), and mid‐July (DOY 199) in 2012, 2013, and 2014, respectively (Figure 2a–h).

**Table 1:** Statistical analysis on the effects of warming (W), N addition (N), year (Y), and measuring date (D) on ecosystem respiration (Reco)

Factors	Reco	
	*F*	*p*
Y	**6.529**	**0.013**
W	**19.800**	**<0.001**
N	2.474	0.055
D	**113.799**	**<0.001**
Y × W	1.654	0.204
Y × N	0.159	0.923
W × N	0.498	0.853
Y × D	**25.358**	**<0.001**
W × D	**5.518**	**<0.001**
N × D	1.357	0.085
Y × W × N	0.132	0.941
Y × W × D	2.362	0.033
Y × N × D	0.966	0.515
W × N × D	0.594	0.983
Y × W × N × D	0.911	0.597

*Note*

The values in bold mean the significant effects.

Warming tended to decrease Reco, but varied with years (Table 1, *p* < 0.001). YW significantly decreased Reco during the 2012 growing season, while in 2013, the effects were not significant (Figure 2a,b). In 2014, compared with NW under the no N addition treatment, WW significantly decreased Reco. Under the N addition treatment, neither YW nor WW had significant effects on Reco (Figure 2c), indicating that N addition compensated the negative effect of warming on Reco.

By averaging daily Reco in the same month, we calculated total C emissions throughout the growing season. The previous study had indicated that C emissions in the growing season accounted for 97.4% of total annual C emissions. We estimated that the total annual C emissions in ambient plots in 2012, 2013, and 2014 were 4,583, 4,082, and 5,532 kg C ha^−1^ year^−1^, respectively (Table 2). Compared with NW, YW and WW decreased annual C emissions, especially in no N addition. YW decreased annual C emissions by 20.1%, 12.5%, and 30.5% in 2012, 2013, and 2014, respectively. WW decreased annual C emissions by 25.1% and 47.1% in 2013 and 2014, respectively (Table 2). Under N treatment, YW and WW had no effect on ecosystem C emissions in 2014 (Table 2).

**Table 2:** Effects of warming and N addition on annual CO_2_ efflux

	NW		YW		WW	
	Mean	*SE*	Mean	*SE*	Mean	*SE*
2012						
CK	4583.55^a^	780.81	3660.31^b^	585.62	NA	NA
N	5321.65^a^	793.89	4327.88^b^	439.34	NA	NA
2013						
CK	4082.02^a^	668.25	3571.72^ab^	764.94	3059.05^b^	571.71
N	4417.54^a^	670.16	3908.72^ab^	864.90	3741.34^b^	541.00
2014						
CK	5532.95^a^	788.53	3840.82^b^	576.81	2926.33^c^	318.46
N	5193.34^a^	705.00	4928.90^a^	706.54	4716.50^a^	520.19

*Notes*

NA represented no available data in WW treatment in 2012.

Different low case letters under the same N addition level represent significant differences among warming treatments.

### 3.3 Plant aboveground and belowground biomass

**Figure 3:**
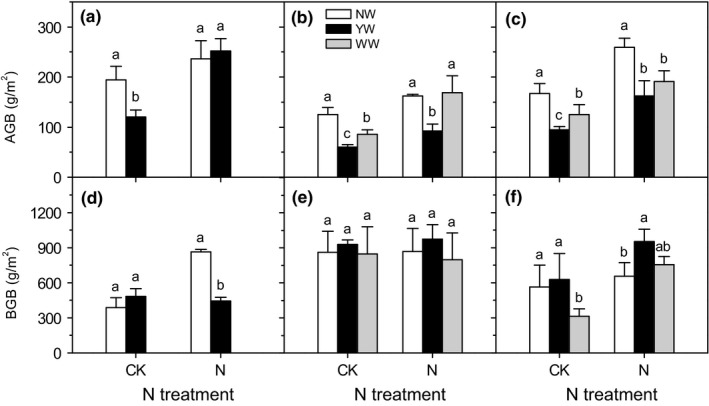
Effects of warming and N addition on plant above‐ (AGB) and belowground biomass (BGB). Different low case letters under the same N addition level represented significant differences among warming treatments

Warming significantly affected aboveground biomass (Table 3, *p* < 0.001). YW and WW significantly decreased aboveground biomass, especially under the no N addition treatment (Figure 3a–c). While under N treatment, only YW decreased aboveground biomass in 2013 (Figure 3b), and both YW and WW significantly decreased aboveground biomass in 2014 (Figure 3c).

**Table 3:** Statistical analysis on the effects of warming (W), N addition (N), and year (Y) on plant above‐ and belowground biomass, soil inorganic N, and microbial biomass C

Factors	Aboveground biomass		Belowground biomass		Soil inorganic N		Soil microbial biomass C	
	*F*	*p*	*F*	*p*	*F*	*p*	*F*	*p*
Y	**32.20**	**<0.001**	**18.62**	**<0.001**	**67.58**	**<0.001**	**17.37**	**<0.001**
W	**19.48**	**<0.001**	2.70	0.083	**30.87**	**<0.001**	**38.63**	**<0.001**
N	**97.22**	**<0.001**	**5.05**	**0.032**	**48.10**	**<0.001**	4.09	0.051
Y × W	**3.153**	**0.037**	1.52	0.226	**5.21**	**0.005**	**3.15**	**0.038**
Y × N	2.408	0.104	1.43	0.253	**7.97**	**0.002**	0.807	0.455
W × N	1.235	0.303	0.734	0.488	2.22	0.125	1.16	0.325
Y × W × N	**4.234**	**0.012**	1.713	0.184	3.30	0.033	0.828	0.488

*Note*

The values in bold mean the significant effects.

The effects of warming on belowground biomass varied with years. Under the N addition treatment, YW only significantly decreased belowground biomass in 2012 (Figure 3d). Under the no N addition treatment, WW decreased belowground biomass in 2014 (Figure 3f). However, under the N addition treatment, YW tended to increase the allocation of biomass to belowground in treatment years (Figure 3e,f).

### 3.4 Soil inorganic N and microbial biomass C

**Figure 4:**
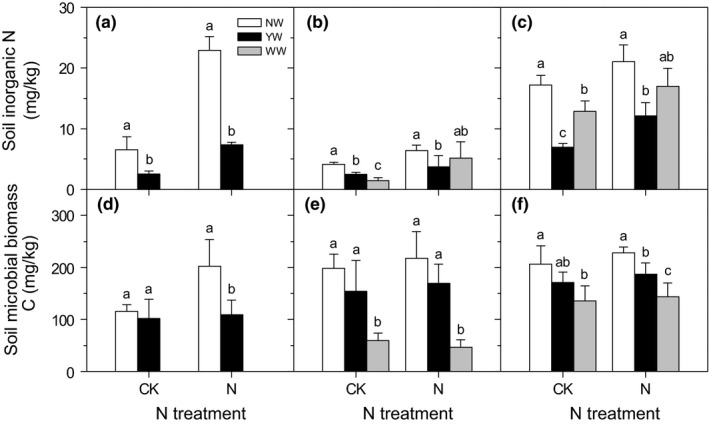
Effects of warming and N addition on soil inorganic N content and soil microbial biomass C. Different low case letters under the same N addition level represented significant differences among warming treatments

Warming significantly affected SIN (Table 3, *p* < 0.001). YW significantly decreased SIN by 61%, 40%, and 60% under no N addition treatment and 68%, 42%, and 42% under N treatment in 2012, 2013, and 2014, respectively (Figure 4a–c). Under no N addition treatment, WW significantly decreased SIN by 65% in 2013 and 25% in 2014. Under N addition treatment, WW had no effect on SIN (Figure 4b,c), which indicated that N addition compensated the SIN decreased by WW.

Warming also significantly affected SMC (Table 3, *p* < 0.001). Under the no N addition treatment, YW had no effects on SMC, while WW significantly decreased SMC by 70% in 2013 and 34% in 2014 (Figure 4d–f). Under the N addition treatment, WW also significantly decreased SMC in the 2013 and 2014 growing seasons, while YW only decreased SMC by 46% in 2012 and 18% in 2014 (Figure 4d–f).

### 3.5 Environmental factors regulating ecosystem respiration

**Figure 5:**
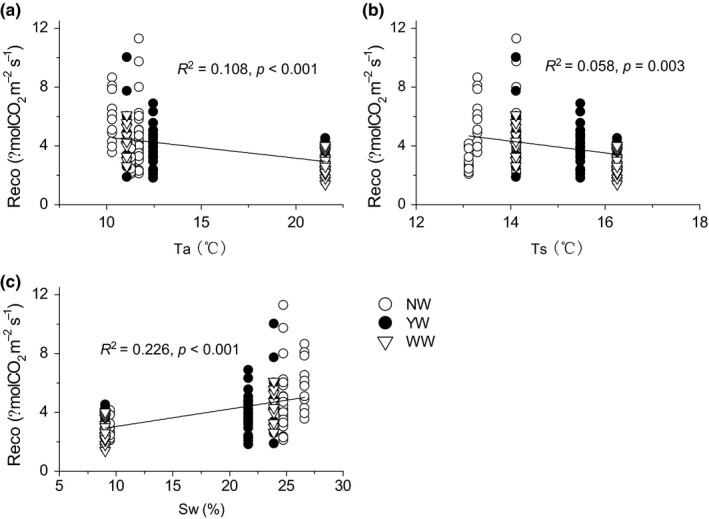
Dependence of ecosystem respiration on air temperature (a), soil temperature (b), and soil water content (c) on interannual scale. Open circles, solid circles, and open triangles represented no warming, year‐round warming, and winter warming treatments, respectively

Regression analysis showed that the seasonal variation of Reco was marginally and negatively correlated with air temperature, and it can explain only 10.8% of the variations of Reco, with soil temperature only explaining 5.8% of the variation of Reco (Figure 5a,b). These correlations indicate that temperature can only explain a small part of the variations of Reco and is not the key controlling factors regulating its seasonal variations. We also found that Reco variation was positively correlated with Sw, with Sw being able to explain 22.6% of its variations (Figure 5c). This finding indicates that, in this semiarid alpine meadow, Sw, rather than temperature, regulates Reco variation and the decrease in Sw in OTC warming devices is the main cause of Reco decline, especially in arid growing seasons.

**Figure 6:**
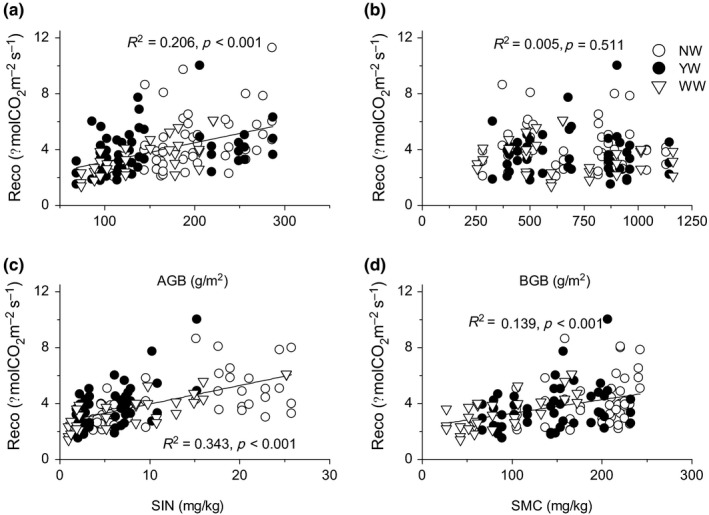
Dependence of ecosystem respiration on aboveground biomass (a), belowground biomass (b), soil inorganic N content (c), and soil microbial biomass C (d) on interannual scales. Open circles, solid circles, and open triangles represented no warming, year‐round warming, and winter warming treatments, respectively

In addition to meteorological factors, plant production and soil properties also regulated the variation of Reco. Regression analysis showed that seasonal variations of Reco were significantly correlated with AGB, SIN, and SMC, while belowground biomass showed no significant correlation with Reco (Figure 6a–d).

**Figure 7:**
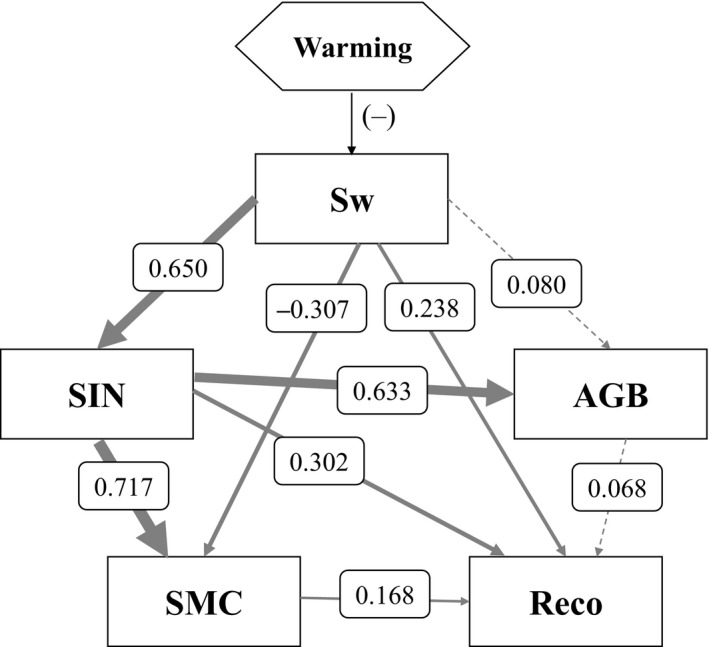
Final structural equation model (SEM) for ecosystem respiration. The thickness of solid arrows reflected the magnitude of the standardized SEM coefficients. Standardized coefficients are listed on each significant path. Nonsignificant paths are showed in dashed lines. Sw, soil inorganic N content, aboveground biomass, soil microbial biomass carbon, and Reco represented soil water content, soil inorganic N content, plant aboveground biomass, soil microbial biomass C, and ecosystem respiration, respectively. The SEM model used in this analysis was *χ* ^2^ = 1.044, probability level = 0.307, RMSEA = 0.019, and CFI = 1.00

Structure equation modeling was further used to explore the relationships between these interactive variables with Reco (Figure 7). The direct, indirect, and total effects of these variables on Reco variation are shown in Table 4. The final model was strong with *χ* ^2^ = 1.044. The chi‐square test showed that our hypothesized path analysis model can be accepted as a potential explanation of the observed covariance matrix (*p* = 0.307). Sw not only had direct effects on Reco (Figure 7, *R* ^2^ = 0.238), but also indirectly affected Reco through influencing soil inorganic N content (SIN) (*R* ^2^ = 0.650) and soil microbial biomass C (SMC) (*R* ^2^ = 0.307). Similarly, SIN not only directly affected Reco (*R* ^2^ = 0.302), but also indirectly affected it through influencing SMC (*R* ^2^ = 0.717). SMC also had a direct effect on Reco (*R* ^2^ = 0.168), while the effect of aboveground biomass (AGB) on Reco was not significant (Figure 7). From the total effects on Reco, Sw was the most important factor affecting Reco (*R* ^2^ = 0.494). SIN and SMC can explain 46.6% and 16.8% of the variations of Reco, while AGB can only explain 6.8% of its variation (Table 4). The results demonstrated that soil properties such as Sw, SIN, and SMC, were key factors regulating the variation of Reco. These findings also indicate that the effects of the changes in warming on soil properties, rather than plant production, affected ecosystem CO_2_ efflux.

**Table 4:** Total direct and indirect effects on Reco in structure equation modeling

Variables	Direct effect	Indirect effect	Total effect
Sw	0.238	0.256	0.494
SIN	0.302	0.164	0.466
SMC	0.168	0.000	0.168
AGB	0.068	0.000	0.068

*Notes*

All the effects were calculated using standardized path coefficients. Sw, soil inorganic N content, aboveground biomass, soil microbial biomass carbon, and Reco represented soil water content, soil inorganic N content, plant aboveground biomass, soil microbial biomass C, and ecosystem respiration, respectively.

## 4 DISCUSSION

Our results demonstrate that the warming treatment significantly increased air and soil temperature and decreased Sw. Warming tended to decrease seasonal and annual Reco, but the extent of the reduction was larger in the WW treatment. The warming and N addition had interactive effects on plant production and soil properties. YW and WW also decreased AGB, SIN, and SMC, especially under the no N addition treatment. While under the N addition treatment, YW significantly decreased SIN and WW significantly decreased SMC. SEM analysis demonstrated that soil properties, such as SWC, SIN, and SMC, were key factors regulating the seasonal and interannual variations of Reco. Winter warming decreased SIN and SMC, which could largely account for the decrease in Reco and subsequent annual C efflux. These findings indicate that, in this alpine meadow, the changes in warming on soil properties rather than plant production had greater effects on ecosystem CO_2_ efflux. In terms of ecosystem C emission, in this semiarid alpine meadow, these results suggest that the effects of winter warming are not as impactful and largely depend on the N deposition rate.

Due to the high latitude, harsh climate, and remote distance of the study site, we mainly collected the data during growing season. In fact, as mentioned above, during growing season, warming altered the plant production and soil properties, and subsequently the ecosystem C efflux. Therefore, the lack of data during the nongrowing season may have effects on an annual timescale. However, plants only generate production in the growing season, and the effects of nongrowing season warming on plant production are manifested in the growing season. In addition, a previous study showed that ecosystem C emissions in the growing season were 97.4% of total annual C emissions (Zhang, 2005). Therefore, the change in ecosystem C efflux in the growing season could largely account for annual timescale change.

### 4.1 Effects of warming on ecosystem C efflux and implication of winter warming

The treatment effects on Reco between the 2013 and 2014 growing seasons differed primarily with respect to soil water availability during these growing seasons. This suggests a high potential for interactive effects between different climate change factors, such as precipitation and climate warming or precipitation and N deposition, increases. These large interannual differences in treatment effects were consistent with the results observed in the previous multiyear simulative global change field experiments (Dukes et al., 2005; Hutchison & Henry, 2010). Neither YW nor WW had significant effects on Reco in 2013 (Figure 4b), a year with a large variation in precipitation, indicating that in this semiarid alpine region, the effects of climate change were substantially regulated by precipitation patterns.

During seasons with rare rainfall events (mostly during the early growing season), warming reduced Reco. Generally, warming promoted N mineralization and provided more N for plant growth, especially in nutrient‐limited ecosystems, but these responses only occurred in the case of sufficient water availability for the plants (Sierra, 1997; de Valpine & Harte, 2001). In semiarid alpine regions, rainfall rarely occurs in the early spring, and during this time period, warming can intensively reduce soil moisture. This reduction resulted in the inhibition of plant growth and thus C emissions. Therefore, the effects of warming on plant growth were more pronounced at the beginning of the growing season, because rare rainfall events reduce soil moisture. The previous study has found that in the semiarid alpine region, soil moisture was an important factor regulating seasonal and large‐scale spatial patterns of Reco (Geng et al., 2012; Jiang et al., 2013). The decline of soil moisture resulting from warming will directly limit ecosystem C emissions. In addition, soil microbial activity and substrate supply could also be inhibited due to the reduction in Sw (Niu et al., 2008; Yan, Chen, Huang, & Lin, 2011).

In the nongrowing season, warming generally promotes N mineralization because of temperature increase (Henry & Jefferies, 2003; Rustad et al., 2001), but under low‐temperature conditions, dormant plants and soil microbes are not actively retaining nutrients. With an increased frequency of soil freeze‐thaw cycles, this may lead to gaseous or leaching soil N loss (Hobbie & Chapin, 1996; Matzner & Borken, 2008; Turner & Henry, 2010). In a previous paper, we also found that the warming treatment significantly decreased SIN in the early growing season (Zong et al., 2013), which would limit plant growth during the following growing season. In addition, another reason for the decrease in Reco in the warming treatment was the inhibition of plant production by warming (Zong et al., 2013). This was determined to occur because plant biomass is an important component of Reco (Jiang et al., 2013; Zong et al., 2013). Furthermore, both Sw decline and soil N loss under warming could cause a decrease in Reco.

Consistent with our hypothesis, there were interactive effects of warming and N addition on Reco, as in the no N and low N addition treatments. On the annual time scale, compared with NW, YW and WW significantly decreased C emissions. Additionally, the results demonstrated that winter warming alone can decrease annual C emissions to the same extent as year‐round warming. This indicates that the earlier start of growing season caused by a warmer winter, not the warming effects over the summertime, was mainly responsible for the overall year‐round effects of warming. The previous studies demonstrated that temperature change under winter warming was one of the most important factors that affected soil N transformation and loss processes (Vidon et al., 2010; Zhou et al., 2011). Under the background of global warming, air temperature change induced by the decrease in winter snow cover in high‐latitude and high‐altitude region could increase the frequency of soil freezing and thawing cycles (Henry, 2008), thereby affecting ecosystem soil C and N cycles and storage (Kreyling, 2010). During the winter, when plant roots are largely in a dormant state, climate warming can increase the N mineralization rate during winter when plant roots are largely in dormant state. Coupled with an increase in soil freeze‐thaw cycles, this may lead to an increase in soil N leaching losses (Hutchison & Henry, 2010; Treat, Wollheim, Varner, & Bowden, 2016), thereby affecting ecosystem production in response to temperature. Climate warming can enhance plant N availability during the growing season by increasing soil N mineralization rates and thus meet the needs of plant production. Further study of climate warming on grassland ecosystem C and N cycle should take into consideration the regulating effects of water availability on key ecosystem processes and the changes in alternating freezing and soil moisture under winter warming. In addition to the effects on climatic factors and soil N availability, winter warming also affects nutrient release to the soil‐by‐soil microorganisms, and this can be verified by winter warming on SMC (Figure 4). The seasonal partitioning of N uptake by plants and soil microorganisms in alpine ecosystems reveals that the soil microbial biomass in active soil layer reaches an annual peak in cold seasons (especially in winter). If then shows a decreasing trend during or shortly after the soil thaw event, concurrently, or followed by, a nutrient pulse that can provide an important nutrient resource for plant growth in the early growing season (Edwards & Jefferies, 2013; Jaeger et al., 1999). As seasonal biogeochemical events, the timing and magnitude of nutrient pulses could be affected by winter warming, which has important implications for ecosystem primary productivity and C efflux under future global change scenarios (Edwards & Jefferies, 2013).

### 4.2 Effects of N addition on ecosystem C efflux

An appropriate quantity of N addition significantly increased ecosystem C emissions, consistent with the effects of N addition on plant production (Zong et al., 2013). In general, due to the high altitudes of alpine ecosystems, the low temperature restricts soil N mineralization, and the soil N content is generally very low, so, for an alpine meadow, soil N availability becomes a key factor limiting production (Bowman, Theodose, Schardt, & Conant, 1993; Cao & Zhang, 2001; Jiang et al., 2013). Exogenous nutrient inputs significantly increased soil nutrient availability, so that leaf N content and photosynthetic capacity increased significantly (Reynold & Thornley, 1982; Lü et al., 2013). A previous study showed that N addition can enhance soil net N mineralization rates (Zong et al., 2013), which would stimulate the decomposition of organic matter in soil, which in turn can improve the soil inorganic N, and lead to an increase in plant production (Wang et al., 2012). An improvement of plant production means more respiration for growth and maintenance (Flanagan & Johnson, 2005), and more photosynthetic products delivered to soil microorganisms (Yan et al., 2011). Therefore, improved plant productivity due to N addition is an important factor in increase in Reco.

The N addition offsets the loss of soil N in the warming treatment, which is more pronounced in the late‐growing season which has many rain events. This is consistent with the results from a study on an old farmland (Hutchison & Henry, 2010). This study found that there were no treatment effects on plant biomass in dry years, while in wet years, warming (both year‐round and winter‐only) combined with N addition approximately doubled plant aboveground productivity, and that these effects were additive (Hutchison & Henry, 2010). This finding indicated that the effect of warming may interact very strongly with interannual variation in precipitation.

### 4.3 Factors regulating ecosystem C efflux on different time scales

Generally, temperature is the most important factor regulating Reco, and the positive correlation between Reco and temperature has been referenced in many ecosystem models (Reichstein et al., 2003; Rey, Petsikos, Jarvis, & Grace, 2005; Zhou, Talley, & Luo, 2009). However, the seasonal dynamic of Reco was less negatively correlated with temperature, but positively correlated with soil moisture (Figure 5), consistent with our previous study (Jiang et al., 2013). The apparent negative effect of soil temperature on ecosystem and soil respiration could be confounded by the effect of the aboveground biomass, especially under nutrient enrichment (Jiang et al., 2013). In semiarid areas, soil moisture plays an important role in regulating the activities of plant production (Niu et al., 2008; Xu & Wan, 2008; Yan et al., 2011) and soil microorganisms (Austin et al., 2004; Bi, Zhang, Liang, Yang, & Ma, 2012). Plant production is the source of the substrate for Reco, and the controlling effects have been verified in many previous studies (Jiang et al., 2013; Yan et al., 2011). Therefore, on a seasonal timescale, the relationship between soil temperature and Reco was confounded by soil moisture (Shen, Li, & Fu, 2015) and plant production (Jiang et al., 2013).

Structure equation modeling analysis demonstrated that soil properties, such as Sw, SIN, and SMC, were key factors regulating the seasonal and interannual variations of Reco. In semiarid areas, soil moisture plays an important role in regulating the activities of plant production (Niu et al., 2008; Xu & Wan, 2008; Yan et al., 2011) and soil microorganisms (Austin et al., 2004; Bi et al., 2012). Sw not only directly affected Reco but also indirectly affected Reco through soil nutrient availability and microbial biomass. Therefore, the decrease in Sw under warming had significant effects on ecosystem C efflux through the subsequent change in soil properties. Soil nutrient availability and microbial biomass also directly or indirectly had effects on Reco (Figure 7). The warming treatment, especially winter warming, decreased SIN and SMC (Figure 4), which could account for the decrease in Reco and subsequently the annual C efflux. As the direct effect of winter warming on soil microorganisms, we also infer that winter warming could affect the timing and magnitude of nutrient pulses. This would have important implications for primary productivity and ecosystem C efflux in alpine ecosystems under future global change scenarios (Edwards & Jefferies, 2013; Jefferies, Walker, Edwards, & Dainty, 2010). In addition, the previous studies showed that soil microbes were the main source of ecosystem C efflux, and the effects of warming on soil microbes can be directly manifested in Reco. Although SIN was also decreased by YW, SIN can only indirectly affect Reco through affecting plant production and other ecological processes. As an available nutrient for plant growth, the increase in soil N availability had significant effects on plant aboveground biomass, while SEM showed that aboveground biomass had only a small effect on Reco variations. These findings indicated that in this semiarid alpine meadow ecosystem, rather than plant production, the changes in warming on soil properties affected ecosystem CO_2_ efflux. They also indicated that the greater effect of winter warming than year‐around warming on ecosystem C efflux can be interpreted by these mechanisms.

Structure equation modeling analysis indicated that in this alpine meadow, the changes in warming on soil property changes, rather than plant production, had greater effects on ecosystem CO_2_ efflux. The results can be interpreted as follows. The proportional change in soil properties induced by warming was larger than plant production. YW significantly decreased SIN by 61%, 40%, and 60% under the no N addition treatment and 68%, 42%, and 42% under the N treatment in 2012, 2013, and 2014, respectively (Figure 4a–c). WW significantly decreased SIN by 65% in 2013 under the no N addition treatment and only decreased AGB by 31% and 25%. The extent of the change in soil properties indicated that warming had larger effects on soil properties change than AGB. Moreover, the previous studies showed that nongrowing season warming has follow‐up effects on ecosystems, as it not only affects the N cycling process during the warming period but also affects the N cycle of subsequent growth seasons and on even longer timescales (Haei, Oquist, Kreyling, Ilstedt, & Laudon, 2013; Mori, Fujii, & Kurokawa, 2014; Turner & Henry, 2010). Therefore, the effects of winter warming on soil properties were direct, while the effects on plant production occurred later and were indirect in growing season. Third, the previous studies also showed that soil microbes were the main source of ecosystem C efflux, and the effects of warming on soil microbes can be directly manifested in Reco. Although SIN was also decreased by YW, SIN only can indirectly affect Reco through its effect on plant production and other ecological processes. Therefore, in this alpine meadow, soil property changes induced by the warming treatment had greater effects on ecosystem CO_2_ efflux.

## 5 CONCLUSION

To our knowledge, this is the first study to evaluate winter warming and separate the effects of warming treatments for ecosystem C efflux and the controlling factors of an alpine meadow on the Qinghai‐Tibetan Plateau. Warming can directly reduce ecosystem CO_2_ emissions by reducing Sw, while winter warming increased SIN loss and decreased SMC, and indirectly affected ecosystem C emissions. N addition could compensate for the decrease in SIN to some extent. The findings indicated that the effects of warming on soil properties are more important than plant production, to affect ecosystem CO_2_ efflux in this semiarid alpine meadow ecosystem. From the aspect of ecosystem C efflux, the effects of winter warming are not as impactful as predicted and largely depend on precipitation pattern and atmospheric N deposition in this semiarid alpine region.

## CONFLICT OF INTEREST

None declared.

## AUTHOR CONTRIBUTIONS

NZ, PLS, and XZZ designed the experiments. NZ, SBG, and CD performed the experiments. NZ, SBG, CD, and XC analyzed the data. NZ, PLS, and XZZ wrote and revised the manuscript.

## DATA ACCESSIBILITY

Data are available from the Dryad Digital Repository: https://doi.org/10.5061/dryad.nq82081.

## References

[ece34484-bib-0001] An, Y. , Wan, S. Q. , Zhou, X. H. , Subedar, A. A. , Wallace, L. L. , & Luo, Y. Q. (2005). Plant nitrogen concentration, use efficiency, and contents in a tallgrass prairie ecosystem under experimental warming. Global Change Biology, 11, 1733–1744.

[ece34484-bib-0002] Austin, A. , Yahdjian, L. , Stark, J. , Belnap, J. , Porporato, A. , Norton, U. , … Schaeffer, S. (2004). Water pulses and biogeochemical cycles in arid and semiarid ecosystems. Oecologia, 141, 221–235. 14986096 10.1007/s00442-004-1519-1

[ece34484-bib-0003] Bi, J. , Zhang, N. , Liang, Y. , Yang, H. , & Ma, K. (2012). Interactive effects of water and nitrogen addition on soil microbial communities in a semiarid steppe. Journal of Plant Ecology, 5, 320–329.

[ece34484-bib-0004] Bowman, W. D. , Theodose, T. A. , Schardt, J. C. , & Conant, R. T. (1993). Constraints of nutrient availability on primary production in two alpine tundra communities. Ecology, 74, 2085–2097.

[ece34484-bib-0005] Cao, G. , & Zhang, J. (2001). Soil nutrition and substance cycle of Kobresia meadow (pp. 58–147). Beijing, China: Science Press.

[ece34484-bib-0006] Chen, H. , Zhu, Q. , Peng, C. , Wu, N. , Wang, Y. , Fang, X. , … Wu, J. (2013). The impacts of climate change and human activities on biogeochemical cycles on the Qinghai‐Tibetan Plateau. Global Change Biology, 19, 2940–2955. 23744573 10.1111/gcb.12277

[ece34484-bib-0007] De Frenne, P. , De Schrijver, A. , Graae, B. J. , Gruwez, R. , Tack, W. , Vandelook, F. , … Verheyen, K. (2010). The use of open‐top chambers in forests for evaluating warming effects on herbaceous understorey plants. Ecological Research, 25, 163–171.

[ece34484-bib-0008] Dormann, C. F. , & Woodin, S. J. (2002). Climate change in the Arctic: Using plant functional types in a meta‐analysis of field experiments. Functional Ecology, 16, 4–17.

[ece34484-bib-0009] Dukes, J. S. , Chiariello, N. R. , Cleland, E. E. , Moore, L. A. , Shaw, M. R. , Thayer, S. , … Field, C. B. (2005). Responses of grassland production to single and multiple global environmental changes. PLoS Biology, 3, 1829–1837. 10.1371/journal.pbio.0030319 PMC1182693 16076244

[ece34484-bib-0010] Edwards, K. A. , & Jefferies, R. L. (2013). Inter‐annual and seasonal dynamics of soil microbial biomass and nutrients in wet and dry low‐Arctic sedge meadows. Soil Biology and Biochemistry, 57, 83–90.

[ece34484-bib-0011] Elser, J. J. , Bracken, M. E. , Cleland, E. E. , Gruner, D. S. , Harpole, W. S. , Hillebrand, H. , … Smith, J. E. (2007). Global analysis of nitrogen and phosphorus limitation of primary producers in freshwater, marine and terrestrial ecosystems. Ecology Letters, 10, 1135–1142. 17922835 10.1111/j.1461-0248.2007.01113.x

[ece34484-bib-0012] Fitzhugh, R. D. , Driscoll, C. T. , Groffman, P. M. , Tierney, G. L. , Fahey, T. J. , & Hardy, J. P. (2001). Effects of soil freezing disturbance on soil solution nitrogen, phosphorus, and carbon chemistry in a northern hardwood ecosystem. Biogeochemistry, 56, 215–238.

[ece34484-bib-0013] Flanagan, L. B. , & Johnson, B. G. (2005). Interacting effects of temperature, soil moisture and plant biomass production on ecosystem respiration in a northern temperate grassland. Agricultural and Forest Meteorology, 130, 237–253.

[ece34484-bib-0014] Galloway, J. N. , Dentener, F. J. , Capone, D. G. , Boyer, E. W. , Howarth, R. W. , Seitzinger, S. P. , … Vöosmarty, C. J. (2004). Nitrogen cycles: Past, present, and future. Biogeochemistry, 70, 153–226.

[ece34484-bib-0015] Geng, Y. , Wang, Y. , Yang, K. , Wang, S. , Zeng, H. , Baumann, F. , … He, J. S. (2012). Soil respiration in Tibetan alpine grasslands: Belowground biomass and soil moisture, but not soil temperature, best explain the large‐scale patterns. PLoS One, 7, e34968. 22509373 10.1371/journal.pone.0034968 PMC3324551

[ece34484-bib-0600] Grace, J. B. (2006). Structural Equation Modeling and Natural Systems. Cambridge University Press.

[ece34484-bib-0016] Groffman, P. M. , Driscoll, C. T. , Fahey, T. J. , Hardy, J. P. , Fitzhugh, R. D. , & Tierney, G. L. (2001). Colder soils in a warmer world: A snow manipulation study in a northern hardwood forest ecosystem. Biogeochemistry, 56, 135–150.

[ece34484-bib-0017] Gruber, N. , & Galloway, J. N. (2008). An earth‐system perspective of the global nitrogen cycle. Nature, 451, 293–296. 18202647 10.1038/nature06592

[ece34484-bib-0018] Haei, M. , Oquist, M. G. , Kreyling, J. , Ilstedt, U. , & Laudon, H. (2013). Winter climate controls soil carbon dynamics during summer in boreal forests. Environmental Research Letters, 8, 024017 10.1088/1748-9326/8/2/024017

[ece34484-bib-0019] Heinemeyer, A. , Di Bene, C. , Lloyd, A. R. , Tortorella, D. , Baxter, R. , Huntley, B. , … Ineson, P. (2011). Soil respiration: Implications of the plant‐soil continuum and respiration chamber collar‐insertion depth on measurement and modelling of soil CO_2_ efflux rates in three ecosystems. European Journal of Soil Science, 62, 82–94.

[ece34484-bib-0020] Henry, H. A. L. (2008). Climate change and soil freezing dynamics: Historical trends and projected changes. Climatic Change, 87, 421–434.

[ece34484-bib-0021] Henry, H. A. L. , & Jefferies, R. L. (2003). Interactions in the uptake of amino acids, ammonium and nitrate ions in the Arctic salt‐marsh grass, *Puccinellia phryganodes* . Plant Cell and Environment, 26, 419–428.

[ece34484-bib-0022] Hobbie, S. E. , & Chapin, F. S. (1996). Winter regulation of tundra litter carbon and nitrogen dynamics. Biogeochemistry, 35, 327–338.

[ece34484-bib-0023] Hutchison, J. S. , & Henry, H. A. L. (2010). Additive effects of warming and increased nitrogen deposition in a temperate old field: Plant productivity and the importance of winter. Ecosystems, 13, 661–672.

[ece34484-bib-0024] IPCC (2007). Intergovernmental Panel on Climate Change (IPCC), Climate change. The Physical Science Basis. The Fourth Assessment Report of Working Group.

[ece34484-bib-0025] Jaeger, C. H. , Monson, R. K. , Fisk, M. C. , & Schmidt, S. K. (1999). Seasonal partitioning of nitrogen by plants and soil microorganisms in an alpine ecosystem. Ecology, 80, 1883–1891.

[ece34484-bib-0026] Jefferies, R. L. , Walker, N. A. , Edwards, K. A. , & Dainty, J. (2010). Is the decline of soil microbial biomass in late winter coupled to changes in the physical state of cold soils? Soil Biology and Biochemistry, 42, 129–135.

[ece34484-bib-0027] Jiang, J. , Zong, N. , Song, M. , Shi, P. , Ma, W. , Fu, G. , … Ouyang, H. (2013). Responses of ecosystem respiration and its components to fertilization in an alpine meadow on the Tibetan Plateau. European Journal of Soil Biology, 56, 101–106.

[ece34484-bib-0028] Joseph, G. , & Henry, H. A. L. (2008). Soil nitrogen leaching losses in response to freeze‐thaw cycle s and pulsed warming in a temperate old field. Soil Biology and Biochemistry, 40, 1947–1953.

[ece34484-bib-0029] Kielland, K. , Olson, K. , Ruess, R. W. , & Boone, R. D. (2006). Contribution of winter processes to soil nitrogen flux in taiga forest ecosystems. Biogeochemistry, 81, 349–360.

[ece34484-bib-0030] Kreyling, J. (2010). Winter climate change: A critical factor for temperate vegetation performance. Ecology, 91, 1939–1948. 20715613 10.1890/09-1160.1

[ece34484-bib-0031] Lamarque, J. F. , Hess, P. , Emmons, L. , Buja, L. , Washington, W. , & Granier, C. (2005). Tropospheric ozone evolution between 1890 and 1990. Journal of Geophysical Research‐Atmospheres, 110, 1–15. 10.1029/2004JD005537

[ece34484-bib-0032] LeBauer, D. S. , & Treseder, K. K. (2008). Nitrogen limitation of net primary productivity in terrestrial ecosystems is globally distributed. Ecology, 89, 371–379. 18409427 10.1890/06-2057.1

[ece34484-bib-0033] Li, L. , Yang, S. , Wang, Z. , Zhu, X. , & Tang, H. (2010). Evidence of warming and wetting climate over the Qinghai‐Tibet Plateau. Arctic Antarctic and Alpine Research, 42, 449–457.

[ece34484-bib-0034] Lin, X. , Zhang, Z. , Wang, S. , Hu, Y. , Xu, G. , Luo, C. , … Xie, Z. (2011). Response of ecosystem respiration to warming and grazing during the growing seasons in the alpine meadow on the Tibetan plateau. Agricultural and Forest Meteorology, 151, 792–802.

[ece34484-bib-0035] Liu, X. , & Chen, B. (2000). Climatic warming in the Tibetan Plateau during recent decades. International Journal of Climatology, 20, 1729–1742.

[ece34484-bib-0036] Lü, X. , Reed, S. , Yu, Q. , He, N. , Wang, Z. , & Han, X. (2013). Convergent responses of nitrogen and phosphorus resorption to nitrogen inputs in a semiarid grassland. Global Change Biology, 19, 2775–2784. 23625746 10.1111/gcb.12235

[ece34484-bib-0037] Luo, Y. , & Zhou, X. (2006). Soil respiration and the environment. London, UK: Academic Press.

[ece34484-bib-0038] Matzner, E. , & Borken, W. (2008). Do freeze‐thaw events enhance C and N losses from soils of different ecosystems? A review. European Journal of Soil Science, 59, 274–284.

[ece34484-bib-0039] Mori, A. S. , Fujii, S. , & Kurokawa, H. (2014). Ecological consequences through responses of plant and soil communities to changing winter climate. Ecological Research, 29, 547–559.

[ece34484-bib-0040] Niu, S. , Wu, M. , Han, Y. , Xia, J. , Li, L. , & Wan, S. (2008). Water‐mediated responses of ecosystem carbon fluxes to climatic change in a temperate steppe. New Phytologist, 177, 209–219. 17944829 10.1111/j.1469-8137.2007.02237.x

[ece34484-bib-0041] Reichstein, M. , Rey, A. , Freibauer, A. , Tenhunen, J. , Valentini, R. , Banza, J. , … Yakir, D. (2003). Modeling temporal and large‐scale spatial variability of soil respiration from soil water availability, temperature and vegetation productivity indices. Global Biogeochemical Cycles, 17, 1104.

[ece34484-bib-0042] Rey, A. , Petsikos, C. , Jarvis, P. G. , & Grace, J. (2005). Effect of temperature and moisture on rates of carbon mineralization in a Mediterranean oak forest soil under controlled and field conditions. European Journal of Soil Science, 56, 589–599.

[ece34484-bib-0043] Reynold, J. F. , & Thornley, J. N. M. (1982). A shoot: Root partitioning model. Annual of Botany, 49, 585–597.

[ece34484-bib-0044] Rustad, L. E. , Campbell, J. L. , Marion, G. M. , Norby, R. J. , Mitchell, M. J. , Hartley, A. E. , … Gcte, N. (2001). A meta‐analysis of the response of soil respiration, net nitrogen mineralization, and aboveground plant growth to experimental ecosystem warming. Oecologia, 126, 543–562. 28547240 10.1007/s004420000544

[ece34484-bib-0045] Schimel, J. P. , Bilbrough, C. , & Welker, J. A. (2004). Increased snow depth affects microbial activity and nitrogen mineralization in two Arctic tundra communities. Soil Biology and Biochemistry, 36, 217–227.

[ece34484-bib-0046] Shen, Z. , Li, Y. , & Fu, G. (2015). Response of soil respiration to short‐term experimental warming and precipitation pulses over the growing season in an alpine meadow on the Northern Tibet. Applied Soil Ecology, 90, 35–40.

[ece34484-bib-0047] Shi, P. , Sun, X. , Xu, L. , Zhang, X. , He, Y. , Zhang, D. , & Yu, G. (2006). Net ecosystem CO_2_ exchange and controlling factors in a steppe‐Kobresia meadow on the Tibetan Plateau. Science in China (Series D:Earth Sciences), 49, 207–218.

[ece34484-bib-0048] Sierra, J. (1997). Temperature and soil moisture dependence of N mineralization in intact soil cores. Soil Biology and Biochemistry, 29, 1557–1563.

[ece34484-bib-0049] Tang, J. , Baldocchi, D. D. , & Xu, L. (2005). Tree photosynthesis modulates soil respiration on a diurnal time scale. Global Change Biology, 11, 1298–1304.

[ece34484-bib-0050] Treat, C. C. , Wollheim, W. M. , Varner, R. K. , & Bowden, W. B. (2016). Longer thaw seasons increase nitrogen availability for leaching during fall in tundra soils. Environmental Research Letters, 11, 064013.

[ece34484-bib-0051] Turner, M. M. , & Henry, H. A. L. (2009). Interactive effects of warming and increased nitrogen deposition on ^15^N tracer retention in a temperate old field: Seasonal trends. Global Change Biology, 15, 2885–2893.

[ece34484-bib-0052] Turner, M. M. , & Henry, H. A. L. (2010). Net nitrogen mineralization and leaching in response to warming and nitrogen deposition in a temperate old field: The importance of winter temperature. Oecologia, 162, 227–236. 19690892 10.1007/s00442-009-1435-5

[ece34484-bib-0053] de Valpine, P. , & Harte, J. (2001). Plant responses to experimental warming in a montane meadow. Ecology, 82, 637–648.

[ece34484-bib-0054] Vance, E. D. , Brookes, P. C. , & Jenkinson, D. S. (1987). An extraction method for measuring soil microbial biomass C. Soil Biology and Biochemistry, 19, 703–707.

[ece34484-bib-0055] Vidon, P. , Allan, C. , Burns, D. , Duval, T. P. , Gurwick, N. , Inamdar, S. , … Sebestyen, S. (2010). Hot spots and hot moments in Riparian Zones, potential for improved water quality management. Journal of the American Water Resources Association, 46, 278–298.

[ece34484-bib-0056] Wang, S. , Duan, J. , Xu, G. , Wang, Y. , Zhang, Z. , Rui, Y. , … Wang, W. (2012). Effects of warming and grazing on soil N availability, species composition, and ANPP in an alpine meadow. Ecology, 93, 2365–2376. 23236908 10.1890/11-1408.1

[ece34484-bib-0057] Wedin, D. A. , & Tilman, D. (1996). Influence of nitrogen loading and species composition on the carbon balance of grasslands. Science, 274, 1720–1723. 8939865 10.1126/science.274.5293.1720

[ece34484-bib-0058] Xu, Z. , Hu, R. , Xiong, P. , Wan, C. , Cao, G. , & Liu, Q. (2010). Initial soil responses to experimental warming in two contrasting forest ecosystems, Eastern Tibetan Plateau, China: Nutrient availabilities, microbial properties and enzyme activities. Applied Soil Ecology, 46, 291–299.

[ece34484-bib-0059] Xu, M. , & Qi, Y. (2001). Soil‐surface CO_2_ efflux and its spatial and temporal variations in a young ponderosa pine plantation in northern California. Global Change Biology, 7, 667–677.

[ece34484-bib-0060] Xu, W. , & Wan, S. (2008). Water‐ and plant‐mediated responses of soil respiration to topography, fire, and nitrogen fertilization in a semiarid grassland in northern China. Soil Biology and Biochemistry, 40, 679–687.

[ece34484-bib-0061] Yan, L. , Chen, S. , Huang, J. , & Lin, G. (2011). Water regulated effects of photosynthetic substrate supply on soil respiration in a semiarid steppe. Global Change Biology, 17, 1990–2001.

[ece34484-bib-0062] Yanai, Y. , Toyota, K. , & Okazaki, M. (2004). Effects of successive soil freeze‐thaw cycles on soil microbial biomass and organic matter decomposition potential of soils. Soil Science and Plant Nutrient, 50, 821–829.

[ece34484-bib-0063] Zhang, D. (2005). The study of ecosystem respiration and the carbon balance in alpine steppe‐meadow on the Tibetan Plateau. Master thesis of the University of Chinese Academy of Sciences.

[ece34484-bib-0064] Zhou, W. , Chen, H. , Zhou, L. , Lewis, B. , Ye, Y. , Tian, J. , … Dai, L. (2011). Effect of freezing‐thawing on nitrogen mineralization in vegetation soils of four landscape zones of Changbai Mountain. Annals of Forest Science, 68, 943–951.

[ece34484-bib-0065] Zhou, X. , Talley, M. , & Luo, Y. (2009). Biomass, litter, and soil respiration along a precipitation gradient in Southern Great Plains, USA. Ecosystems, 12, 1369–1380.

[ece34484-bib-0066] Zong, N. , Chai, X. , Shi, P. , & Yang, X. (2018). Effects of warming and nitrogen addition on plant photosynthate partitioning in an alpine meadow on the Tibetan Plateau. Journal Plant Growth Regulation, 37, 803–812. 10.1007/s00344-017-9775-6

[ece34484-bib-0067] Zong, N. , Shi, P. , Chai, X. , Jiang, J. , Zhang, X. , & Song, M. (2017). Responses of ecosystem respiration to nitrogen enrichment and clipping mediated by soil acidification in an alpine meadow. Pedobiologia, 60, 1–10.

[ece34484-bib-0800] Zong, N. , Shi, P. , Song, M. , Zhang, X. , Jiang, J. , & Chai, X. (2016). Nitrogen critical loads for an alpine meadow ecosystem on the Tibetan Plateau. Environmental Management, 57, 531–542. 26475686 10.1007/s00267-015-0626-6

[ece34484-bib-0068] Zong, N. , Shi, P. , Jiang, J. , Song, M. , Xiong, D. , Ma, W. , … Shen, Z. (2013). Responses of ecosystem CO_2_ fluxes to short‐term experimental warming and nitrogen enrichment in an alpine meadow, northern Tibet Plateau. Scientific World Journal, 2013, 1–11. Article ID 415318. 10.1155/2013/415318 PMC3891229 24459432

[ece34484-bib-0069] Zong, N. , Song, M. , Shi, P. , Jiang, J. , Zhang, X. , & Shen, Z. (2014). Timing patterns of nitrogen application alter plant production and CO_2_ efflux in an alpine meadow on the Tibetan Plateau, China. Pedobiologia, 57, 263–269.

